# Characterizing the role of atrial natriuretic peptide signaling in the development of embryonic ventricular conduction system

**DOI:** 10.1038/s41598-018-25292-0

**Published:** 2018-05-02

**Authors:** Arun Govindapillai, Adam Hotchkiss, Mark Baguma-Nibasheka, Robert A. Rose, Lucile Miquerol, Oliver Smithies, Nobuyo Maeda, Kishore B. S. Pasumarthi

**Affiliations:** 1 Department of Pharmacology, Dalhousie University, Halifax, Nova Scotia Canada; 2 Department of Physiology, Dalhousie University, Halifax, Nova Scotia Canada; 3 IBDM, Aix-Marseille Université, CNRS UMR 7288 Marseille, France; 4 Department of Pathology and Laboratory Medicine, University of North Carolina, Chapel Hill, USA

## Abstract

Patients born with congenital heart defects frequently encounter arrhythmias due to defects in the ventricular conduction system (VCS) development. Although recent studies identified transcriptional networks essential for the heart development, there is scant information on the mechanisms regulating VCS development. Based on the association of atrial natriuretic peptide (ANP) expression with VCS forming regions, it was reasoned that ANP could play a critical role in differentiation of cardiac progenitor cells (CPCs) and cardiomyocytes (CMs) toward a VCS cell lineage. The present study showed that treatment of embryonic ventricular cells with ANP or cell permeable 8-Br-cGMP can induce gene expression of important VCS markers such as hyperpolarization-activated cyclic nucleotide-gated channel-4 (HCN4) and connexin 40 (Cx40). Inhibition of protein kinase G (PKG) via Rp-8-pCPT-cGMPS further confirmed the role of ANP/NPRA/cGMP/PKG pathway in the regulation of HCN4 and Cx40 gene expression. Additional experiments indicated that ANP may regulate VCS marker gene expression by modulating levels of miRNAs that are known to control the stability of transcripts encoding HCN4 and Cx40. Genetic ablation of NPRA revealed significant decreases in VCS marker gene expression and defects in Purkinje fiber arborisation. These results provide mechanistic insights into the role of ANP/NPRA signaling in VCS formation.

## Introduction

The cardiac conduction system (CCS) is a complex network of cells within the heart that generates and conducts electrical impulses to enable rhythmic, coordinated contraction of the heart^1^. The main components of CCS are SA node, AV node, bundle of His, bundle branches and Purkinje fibers. The bundle of His, bundle branches and Purkinje fibers are referred to as the ventricular conduction system (VCS). Availability of various lineage tracking mouse models has increased our understanding of heart development, however, the mechanisms regulating VCS development are not very well characterized^2^. There is evidence that paracrine factors secreted from the coronary endothelium and endocardium [e.g. endothelin-1 (ET-1), neuregulin-1 (Nrg-1)] provide instructive cues for the VCS cell fate^1–3^. ET-1 treatment was shown to increase the proportion of Purkinje cell to cardiomyocyte (CM) ratio in embryonic chick ventricular myocyte cultures^1^. Additional studies demonstrated that Nrg-1 can induce embryonic mouse CMs to differentiate into cells of the conduction system^3^. While these studies suggest conversion of CMs into VCS cells, other studies suggest the existence of a common progenitor cell for working CM and VCS cells^4–6^. Although ET-1 has been shown to play a critical role in the development of VCS in chick heart development^1^, mice lacking ET-1 receptors were viable and they did not reveal any VCS abnormalities^7^. Interestingly, Nrg-1 but not ET-1 treatment increased the expression of a VCS specific reporter gene expression in E9.5 mouse embryos cultured for 48 hrs^3^. Inability of Nrg-1 to increase reporter gene expression in E10.5/11.5 hearts^3^ suggests that additional factors may be involved in the development and/or maturation of VCS network in later stages of heart development.

Atrial natriuretic peptide (ANP) is a paracrine factor and a member of the natriuretic peptide family, involved in regulating cardiovascular homeostasis^8^. ANP is expressed in the primitive heart tube by E8.5 and subsequently down regulated in the murine ventricular chambers by E15 while maintaining high levels in the atria throughout development^9^. However, ANP expression persists after E15 stage in some ventricular cells which are destined to form the VCS^10–13^. These observations suggest that ANP may be involved in the induction and or maturation of the VCS cells. It was shown that exogenous addition of ANP was associated with reduced rates of cardiac progenitor cell (CPC) proliferation, and that ANP-rich regions of the trabecular myocardium were characterized by a lower index of proliferation compared to the adjacent compact layer^14^. Since trabeculae are known to house VCS progenitor cells^2,10,15^, it was speculated that reduced CPC proliferation mediated by ANP/NPRA signaling could be coupled to recruitment of these cells into the conduction system lineage^14^. The present study examined the potential impact of ANP on formation of the VCS cells in the embryonic mouse heart. Our results revealed that ANP induces gene expression of important VCS markers such as hyperpolarization-activated cyclic nucleotide-gated channel-4 (HCN4) and connexin 40 (Cx40) in E11.5 ventricular cell cultures which are known to harbor both CPCs and CMs^5,16,17^, through the natriuretic peptide receptor-A (NPRA) signaling pathway. Pharmacological inhibition as well as genetic ablation of NPRA revealed significant decreases in VCS marker gene expression and defects in Purkinje fiber arborisation.

## Results

### Effects of ANP on percent distribution of cells expressing HCN4 and Cx40 in E11.5 mouse ventricular cell cultures

**Figure 1:**
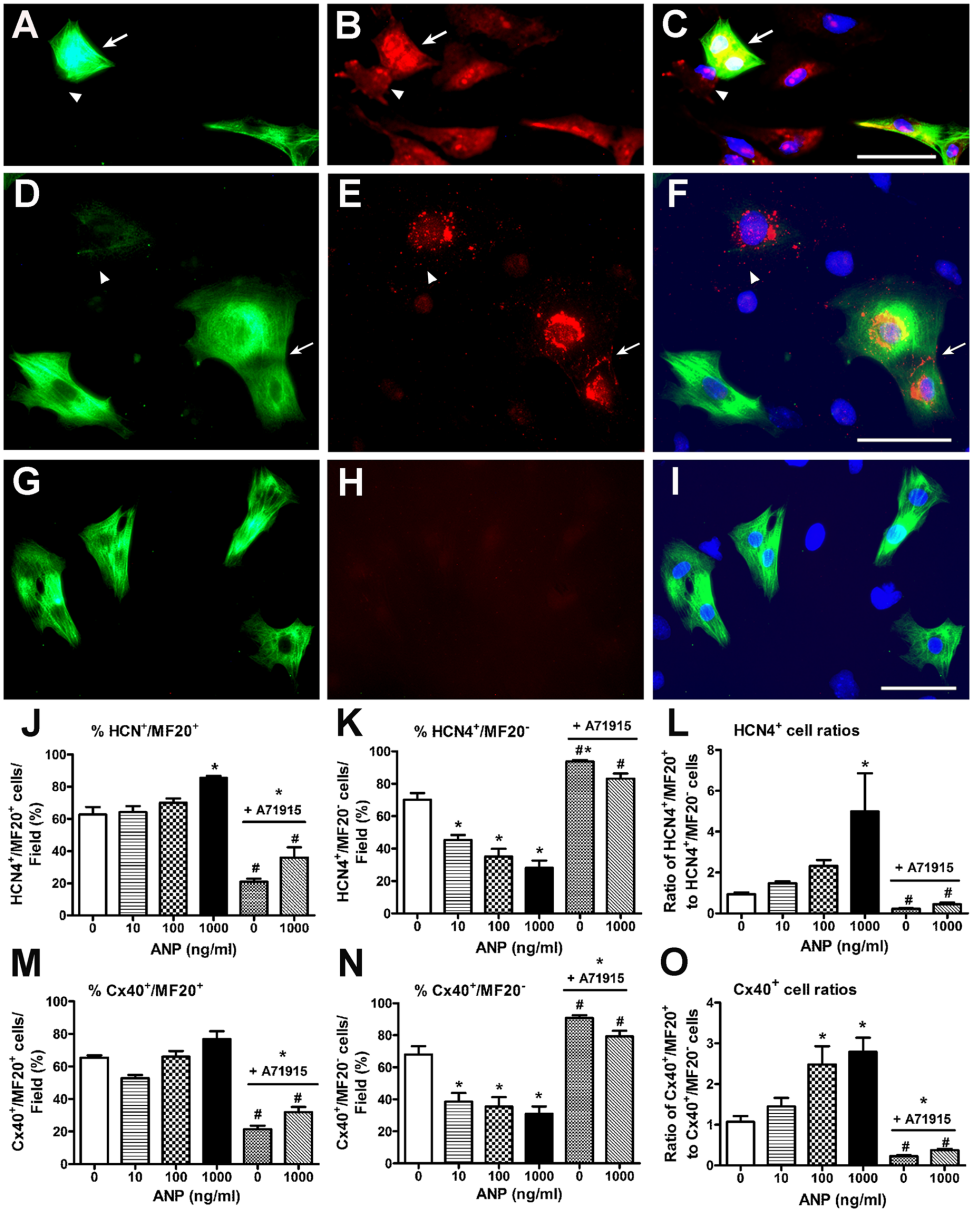
Quantification of HCN4 and Cx40 positive cells in E11.5 mouse ventricular cell cultures following addition of exogenous ANP and or NPRA inhibitor. (**A**–**C**) Representative images of cells co-stained with sarcomeric myosin, MF20 (A) and HCN4 (B) antibodies along with Hoechst nuclear stain (C). (**D**–**F**) Cells co-stained with MF20 (D) and Cx40 (E) antibodies. (**G–I**) Cells were co-stained with MF20 (G) and rabbit nonimmune IgG (H) antibodies. Overlays of immunostaining signals with Hoechst staining were shown in panels C, F and I. Arrows indicate HCN4+ or Cx40+/MF20+ cardiomyocytes, arrow-heads indicate HCN4+ or Cx40+/MF20− nonmyocytes/VCS progenitor cells. A-I: scale bars = 50 μM. (**J–O**) Percentage distribution and ratios of HCN4+/MF20+ and HCN4+/MF20− cells (J–L) or Cx40+/MF20+ and Cx40+/MF20− cells (**M**–**O**) in E11.5 mouse ventricular cell cultures treated with ANP (0, 10, 100 and 1000 ng/ml) and or NPRA inhibitor A71915 (1 µM). N = 5 independent experiments, ~600–900 cells were counted for each group. Each bar represents mean ± SEM, *P < 0.05 Vs. 0 ng/ml (control), ^#^*P* < 0.05 Vs. ANP (1000 ng or 1 µg/ml) for each panel in J–O, One-way ANOVA with Tukey’s multiple comparisons post hoc test.

HCN4 and Cx40 are expressed in the developing CCS^18–20^. To determine whether ANP plays any role in the induction of HCN4 and Cx40 expression, exogenous ANP was added at varying concentrations (0–1000 ng/ml) to primary cultures prepared from E11.5 CD1 ventricles at 12 hr intervals for 48hrs. Cells were fixed and processed for immunostaining with antibodies specific for sarcomeric myosin (MF20) and HCN4 or Cx40. Expression of HCN4 or Cx40 was observed in both MF20 positive (MF20+) or MF20 negative (MF20−) cells and immunostaining patterns revealed cytoplasmic, perinuclear and membrane localizations for both markers (Fig. 1A–F). In control experiments, cells co-stained with MF20 and rabbit nonimmune IgGs did not show any staining in both MF20+ and MF20− cells (see panel H in Fig. 1G–I).

The addition of ANP at 1000 ng/ml (1 µg/ml) but not at lower concentrations resulted in a significant increase in the percentage of HCN4+/MF20+ cells when compared to that of control cultures (1.4-fold, *P* < 0.05, Fig. 1J). In contrast, the percentage of HCN4+/MF20− cells significantly decreased with all concentrations of ANP tested (Fig. 1K). The addition of A71915 (an NPRA antagonist) to E11.5 ventricular cells significantly reduced the percentage of HCN4+/MF20+ cells (3-fold) and increased the percentage of HCN4+/MF20− cells (1.4-fold) compared to control cultures (*P* < 0.05, Fig. 1J,K). To determine if ANP could rescue the effects of NPRA inhibitor, a combination of ANP (1 µg/ml) and A71915 (1 µM) were added to ventricular cells. This combination treatment also led to a significant decrease in the percentage of HCN4+/MF20+ cells (1.8-fold) with no significant effect on the percentage of HCN4+/MF20− cell population when compared to control cultures (Fig. 1J,K). Furthermore, the ratio of HCN4+/MF20+ to HCN4+/MF20− cells significantly increased with 1 μg/ml ANP treatment, while the cell ratios decreased significantly with A71915 treatment in the presence or absence of exogenous ANP (Fig. 1L).

Similarly, the percentages of MF20+ or MF20− cells that were also positive for Cx40 immunostaining were determined upon addition of varying concentrations of exogenous ANP, A71915 alone, or a combination of A71915 and ANP (Fig. 1M,N). Although there was no significant difference in the percentage of Cx40+/MF20+ cells, the percentage of Cx40+/MF20− cells significantly decreased with all concentrations of ANP tested (Fig. 1M,N). Addition of A71915 to E11.5 ventricular cells significantly decreased the percentage of Cx40+/MF20+ cells (3-fold) and increased the percentage of Cx40+/MF20− cells (1.4-fold) when compared to control cultures (Fig. 1M,N). Addition of ANP (1 μg/ml) in combination with A71915 (1 µM) revealed similar effects on the percentages of Cx40+/MF20+ and Cx40+/MF20− cells when compared to the A71915 treatment alone (Fig. 1M,N). Furthermore, the ratio of Cx40+/MF20+ to Cx40+/MF20− cells significantly increased with 1 μg/ml ANP treatment, while the cell ratios decreased significantly with A71915 treatment in the presence or absence of exogenous ANP (Fig. 1O). Collectively, these results indicate that ANP/NPRA signaling can modulate the relative distribution of HCN4 or Cx40 positive cell fractions within the embryonic ventricular cell cultures.

### Gene expression analysis of HCN4 and Cx40 in E11.5 mouse ventricular cells following exogenous ANP treatment

**Figure 2:**
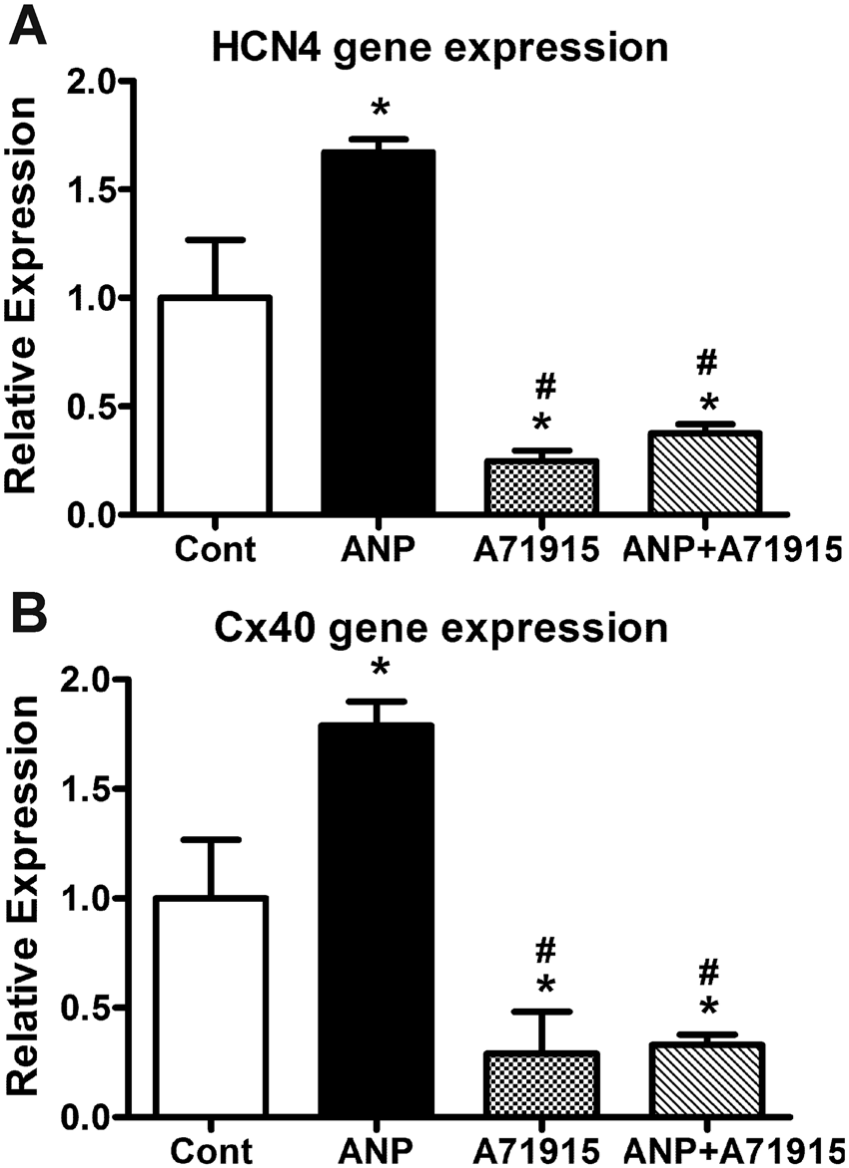
Gene expression of conduction system markers in E11.5 cultures treated with or without ANP and A71915. RT-qPCR analysis was used to monitor HCN4 (**A**) and Cx40 (**B**) gene expression levels in cells treated with ANP (1 μg/ml) and or NPRA inhibitor, A71915 (1 µM) for 48 hours. Expression levels were normalized to GAPDH via ∆∆C_T_ method. Relative expression levels were presented as fold changes in comparison to the levels in untreated cultures (Cont). N = 6 experiments per group, each bar represents mean ± SEM, *P < 0.05 Vs. Cont, ^#^*P* < 0.005 Vs. ANP (1 μg/ml) for both panels, One-way ANOVA with Tukey’s multiple comparisons post hoc test.

Since 1 μg/ml of ANP and 1 µM of A71915 were effective in modulating the ratios of MF20+ to MF20− cells that were also positive for either HCN4 or Cx40, these concentrations were used for subsequent experiments in the present study. For gene expression studies, cultures were treated with or without ANP and A71915 for 48hrs as described earlier. Total RNA was isolated and processed for RT-qPCR analysis using primers specific for HCN4 and Cx40 (Suppl. Table S1) and gene expression levels were normalized to the housekeeping gene glyceraldehyde 3-phosphate dehydrogenase (GAPDH) levels via ∆∆C_T_ method^21^ as described earlier^14,22^. Exogenous addition of ANP to E11.5 ventricular cells resulted in significant increases in gene expression of HCN4 (1.7-fold) and Cx40 (1.8-fold) when compared to the gene expression levels in control cells (*P* < 0.05, Fig. 2A,B). Addition of A71915 significantly reduced gene expression of both HCN4 and Cx40 when compared to control levels (4-fold and 3.4-fold respectively Vs. control, *P* < 0.05, Fig. 2A,B). The combination of ANP and A71915 treatment also resulted in a significant decrease in both HCN4 and Cx40 gene expression (2.7-fold and 3-fold Vs. control, *P* < 0.05, Fig. 2). These results suggest that changes in HCN4 and Cx40 gene expression levels can account for the modulation of HCN4+ and Cx40+ cell percentages after exogenous treatment with ANP and A71915.

### The effects of ANP and A71915 on intracellular cGMP production in E11.5 ventricular cells

**Figure 3:**
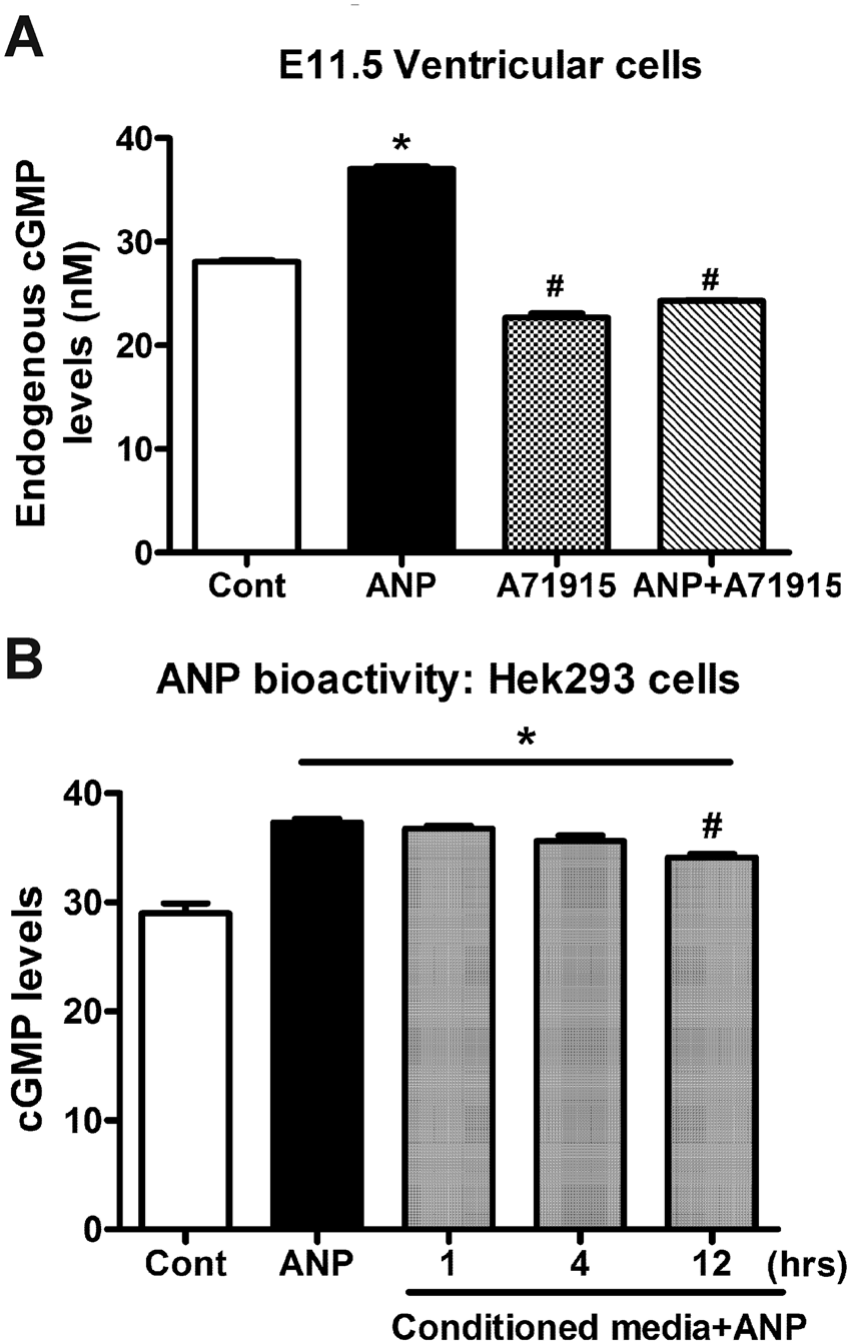
The effects of exogenous ANP and A71915 on cGMP production in E11.5 ventricular cells and assessment of ANP bioactivity. (**A**) Cellular cGMP levels in E11.5 ventricular cells treated with ANP (1 μg/ml) and or A71915 (1 µM) were determined using a competitive HTRF immunoassay. The baseline level of cGMP (Cont) was measured to be 28.1 ± 0.2 nM/ 100,000 cells. Addition of exogenous ANP resulted in a significant increase in cGMP. Co-treatment of cells with both ANP and A71915 resulted in lower levels cGMP compared to untreated cells. N = 3 independent experiments, performed in duplicate wells. Each bar represents mean ± SEM. *P < 0.005 Vs. Cont, ^#^*P* < 0.005 Vs. Cont or ANP (1 μg/ml), One-way ANOVA with Tukey post hoc test. (**B**) Assessment of ANP bioactivity in the conditioned media of E11.5 ventricular cells: Conditioned media was collected at 1, 4 and 12-hour time points and transferred to HEK293 cells, and cGMP levels were measured. As a negative control (Cont), cGMP production was measured in HEK293 cells that were not treated with ANP. As a positive control, cGMP levels were measured in HEK293 cells treated directly with fresh ANP (1 μg/ml). Over 12 hours, conditioned media treatment maintained significantly higher levels of cGMP with a minimal decline in bioactivity. N = 4 independent experiments, performed in duplicate wells. Each bar represents mean ± SEM. *P < 0.005 Vs. Cont, ^#^p < 0.05 Vs. ANP or 1 hr, One-way ANOVA with Tukey post hoc test.

Significant decreases in gene expression of HCN4 and Cx40 with NPRA blocker alone warranted further assessment of A71915 effects on intracellular cGMP levels in embryonic ventricular cells. To determine the effects of ANP and A71915 on second messenger levels in E11.5 ventricular cells, a competitive HTRF immunoassay was performed and intracellular cGMP levels were obtained using a standard curve from the known cGMP concentrations as described earlier^14^. Addition of exogenous ANP significantly increased intracellular cGMP Vs. control (37.4 ± 0.2 nM Vs. 28.1 ± 0.2 nM, *P* < 0.005, Fig. 3A). A71915 alone and the combination of A71915 and ANP were also added to determine the effects on cGMP production. Notably, A71915 treatment alone significantly decreased cGMP levels in these cells when compared to control or ANP treated cells (22.7 ± 0.4 nM, *P* < 0.005, Fig. 3A). Similarly, there was a significant decrease in cGMP production in cells treated with combination of ANP and A71915 (24.3 ± 0.1 nM, Fig. 3A). These results suggest that A71915 can act as an inverse agonist by decreasing intracellular cGMP levels compared to the untreated cells.

### Determination of the bioactivity of exogenously added ANP in embryonic ventricular cell cultures

Secreted ANP is known to be internalized via NPRC mediated clearance mechanism and also subjected for degradation by neutral endopeptidases^8^. To measure the bioactivity of exogenously added ANP, conditioned media supplemented with ANP (1 μg/ml) was collected from E11.5 ventricular cell cultures at 1, 4 or 12 hr duration and transferred to wells containing HEK293 cells which are known to express NPRA receptors^23^. Subsequently, HEK293 cells were incubated at 37 °C for 1 hr and intracellular cGMP levels were measured using a competitive immunoassay. As additional controls, HEK293 cells were treated with or without ANP (1 μg/ml) in fresh medium for comparisons. Incubation of HEK293 cells with fresh medium supplemented with ANP significantly increased cGMP levels compared to untreated cells (37.3 ± 0.2 nM Vs. 29 ± 0.9 nM, *P* < 0.005, Fig. 3B). Furthermore, conditioned media samples retained significantly higher bioactivity compared to untreated cells at all time points tested (Fig. 3B). Although there was a slight but significant decrease in the bioactivity at 12 hr time point compared to that from the 1 hr time point, ANP was still bioactive after 12hrs since it significantly increased cGMP levels compared to untreated cells (34.1 ± 0.4 nM Vs. 29 ± 0.9 nM, *P* < 0.005; Fig. 3B).

### Effects of ANP on Cx40 reporter gene expression in E11.5 Cx40^egfp^ ventricles

**Figure 4:**
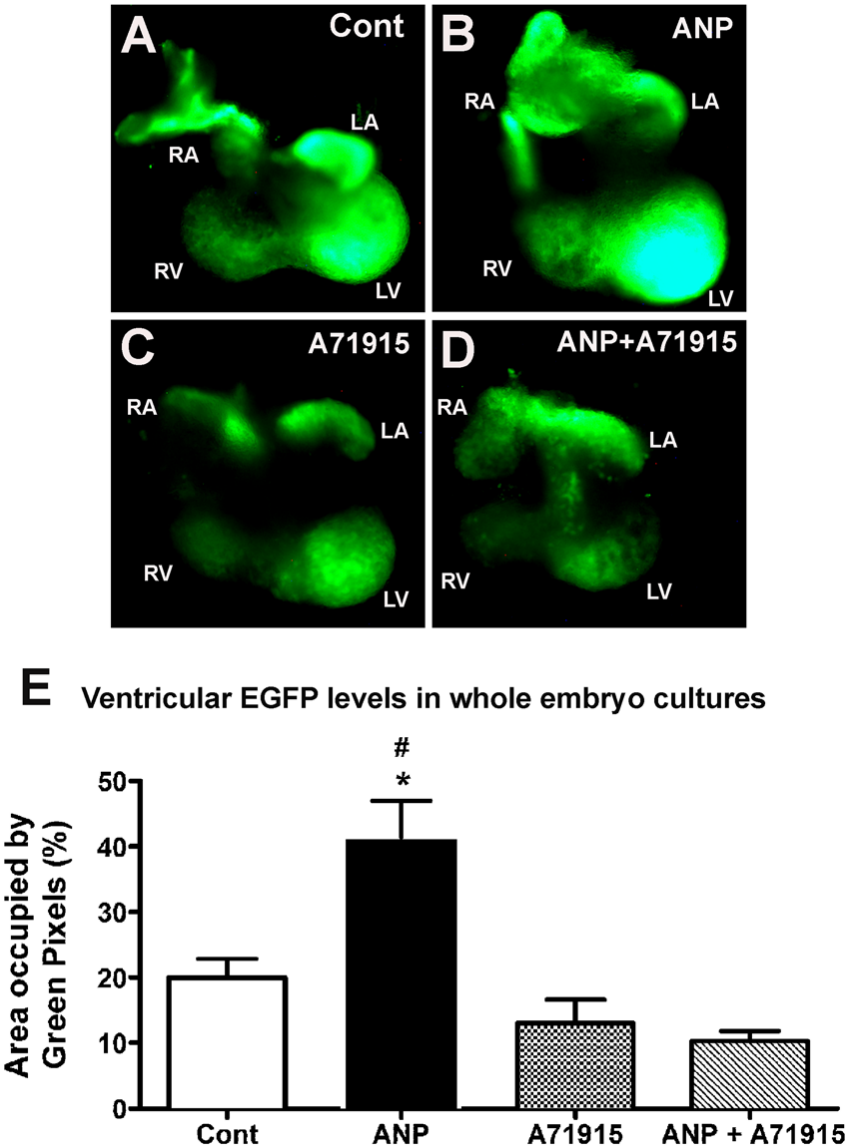
The effects of exogenous ANP and A71915 on EGFP reporter fluorescence in whole embryo cultures derived from Cx40^egfp^ mice. (**A**–**D**) At E10.5, Cx40^egfp/+^ whole embryos were cultured in the presence or absence of ANP (1 μg/ml) and/or A71915 (1 µM) for 24 hours. After 24 hours, whole hearts were isolated at E11.5 stage and reporter gene expression was visualized. ANP-treated whole hearts had strong EGFP signals in both atria and ventricles, with the strongest signal coming from the left ventricle. A71915-treated hearts and combination-treatment hearts showed weaker EGFP signals in all four chambers of the heart. RA: right atrium, RV: right ventricle, LA: left atrium, LV: left ventricle and OFT: outflow tract. (**E**) Quantification of green pixels (%) to determine reporter gene expression in Cx40^egfp+/−^ ventricles at E11.5 treated with ANP and/or A71915. N = 5 hearts per group. Each bar represents mean ± SEM. *P < 0.05 Vs. Cont, ^#^*P* < 0.005 Vs. A71915 or ANP + A71915. One-way ANOVA with Tukey post hoc test.

To further evaluate the role of ANP in Cx40 gene expression, we have employed a Cx40^efgp^ reporter mouse model^24^. Homozygous Cx40^egfp/egfp^ knock-in mice were bred to wild type (WT) C57/BL6 mice to generate heterozygous embryos (Cx40^egfp/+^) at E10.5. Whole embryo cultures were treated with ANP (1 μg/ml) and/or A71915 (1 µM) for 24 hours. Subsequently, embryonic hearts at E11.5 were imaged under fluorescence microscopy (Fig. 4A–D). Compared to the control samples, the hearts treated with ANP revealed strong EGFP signals in both the atrial chambers and ventricles, with the strongest signal in the left ventricle (Fig. 4A,B, N = 5 hearts per group). A71915 treated hearts had very weak EGFP signals in both atria and ventricles and in 2 out of 5 hearts, the atrial fluorescence was barely visible (Fig. 4C). In hearts treated with the combination of ANP and A71915, both atria and ventricles had very weak EGFP signals as well (Fig. 4D). We quantified the percent area occupied by the green pixels in both left and right ventricles of each heart treated with or without ANP and A71915. Compared to the control ventricles, ANP treated ventricles had a significantly larger area of green pixels (2-fold, *P* < 0.05; Fig. 4E). This area was also significantly greater than that of ventricles treated with either A71915 alone or ANP + A71915 combination (*P* < 0.005; Fig. 4E). The addition of A71915 alone or combination treatment resulted in ventricles with smaller areas of EGFP signal compared to control ventricles (1.5- to 1.9-fold), however, there was no statistical significance when the areas occupied by green pixels were compared between groups (*P* = NS). Therefore, these results suggest that the addition of ANP can significantly increase reporter gene fluorescence signal in Cx40^egfp^ hearts at E11.5 stage.

### Expression of conduction system marker genes in ventricles of E14.5 embryos lacking NPRA

**Figure 5:**
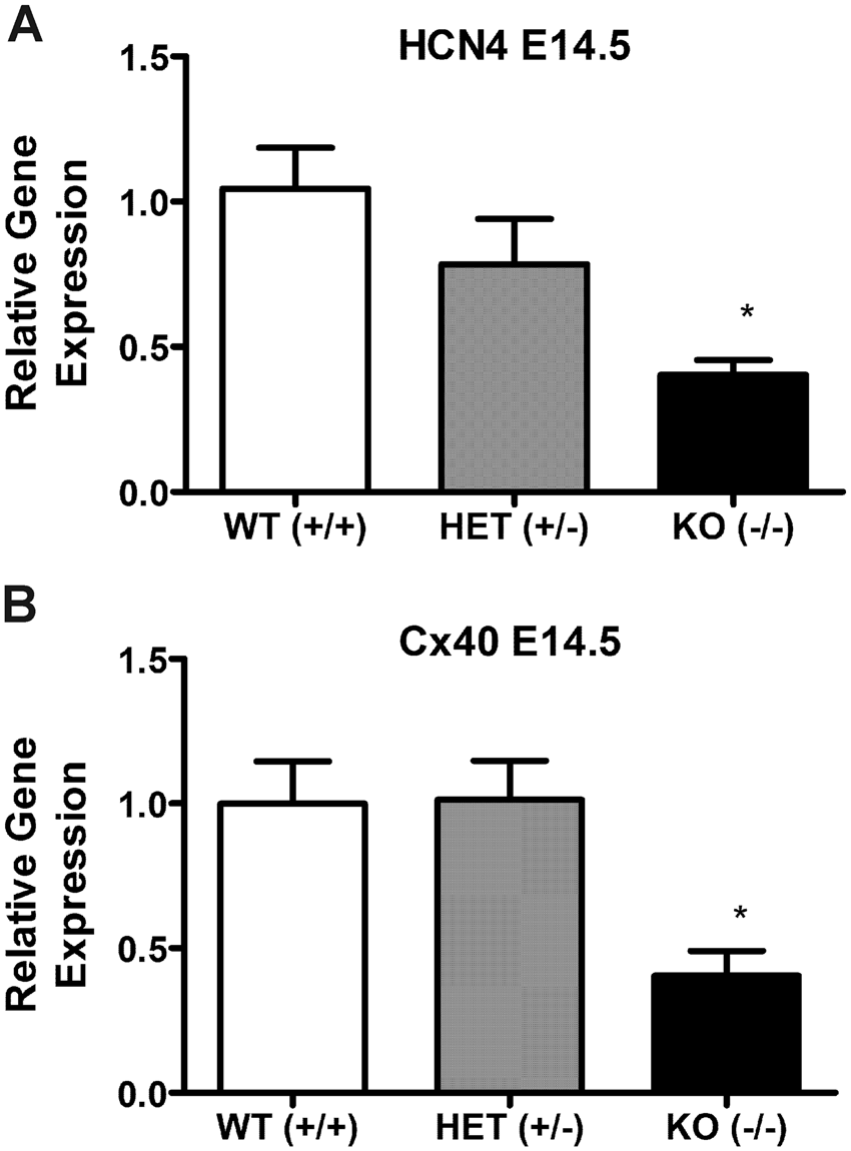
Gene expression of HCN4 and Cx40 in NPRA wild type (WT), heterozygous (HET) and knockout (KO) ventricles at E14.5 stage. RT-qPCR analysis was used to monitor HCN4 (**A**) and Cx40 (**B**) gene expression levels in the ventricles of indicated NPRA genotypes. Expression levels were normalized to GAPDH via ∆∆C_T_ method. Relative expression levels were presented as fold changes in comparison to the levels in WT. N = 8–13 independent RNA extractions/genotype for both panels, each bar represents mean ± SEM, *P < 0.05 Vs. WT for HCN4 gene expression and *P < 0.05 Vs. WT or HET for Cx40 gene expression. One-way ANOVA with Tukey’s multiple comparisons post hoc test.

As an alternative approach to assessing the involvement of ANP/NPRA signaling in VCS development, we employed a previously established NPRA knockout (KO) mouse model^25^. We extracted total RNA from the ventricles of homozygous and heterozygous NPRA KO as well as WT littermates at E14.5 stage and conducted RT-qPCR analysis for HCN4 and Cx40 gene expression levels. E14.5 stage was selected due to difficulties with obtaining sufficient number of ventricles from early developmental stages for each genotype. While the gene expression of HCN4 in NPRA heterozygous ventricles (+/−) revealed a decreasing trend compared to that in WT ventricles (+/+), HCN4 expression levels significantly decreased in homozygous ventricles (−/−) when compared to those in WT samples (2.6-fold reduction Vs. WT, *P* < 0.05, Fig. 5A). Gene expression of Cx40 in heterozygous ventricles was similar to that in WT samples, however, the homozygotes had significantly less Cx40 gene expression compared to WT or heterozygous ventricles (*P* < 0.05, 2.5-fold reduction, Fig. 5B).

### Visualization and quantification of VCS development in newborn mice lacking NPRA using the Cx40^egfp^ mouse model

**Figure 6:**
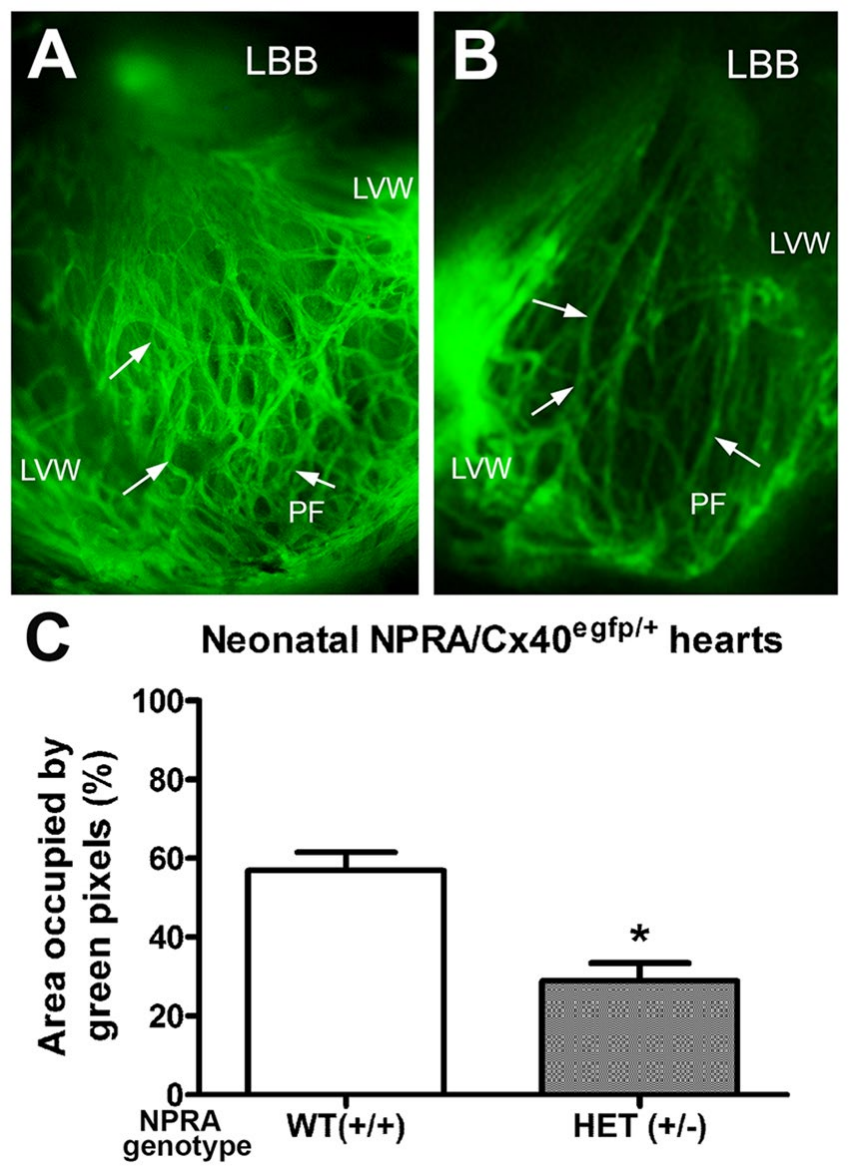
Quantification of ventricular conduction system development in neonate day 1 (ND1) ventricles of wild type (WT) and NPRA^+/−^ mice using a Cx40^egfp+^ reporter approach. (**A**) In the left ventricles (LV) of Cx40^egfp/+^/NPRA^+/+^ ND1 pups, the left bundle branch (LBB) was continuous with an extensively arborized Purkinje fiber (PF) network. Note the typical honeycomb pattern of PFs (arrows) in the septal surface and left and ventricular free wall (LVW). (**B**) In the LV of Cx40^egfp/+^/NPRA^+/−^ pups, PF network appeared to be sparse with fewer egfp positive PF fibers (arrows) within the septal surface and LVW. N = 8–10 NDl pups per genotype. (**C**) Quantification of EGFP green pixels in ND1 ventricles of WT and NPRA^+/−^ mice. *P < 0.005, Student’s unpaired t-test.

The Cx40^egpf^ mouse model permits visualization of His-Purkinje system^15,24^ and has been widely used to monitor development and maturation of VCS^26,27^. In this study, homozygous Cx40^egfp/egfp^ mice were initially bred with NPRA^+/−^ mice to generate compound Cx40^egfp/+^/ NPRA^+/−^ offspring. Subsequent crosses between these F1 progeny were performed to obtain F2 newborn pups harboring a single copy of EGFP (Cx40^egfp/+^) and a variable copy number of the NPRA gene. Neonatal day 1 (ND1) stage was selected for these studies because VCS arborisation can be readily visualized in the ventricles. Thus far, these breeding schemes have yielded Cx40^egfp/+^ ND1 pups that were also NPRA WT (NPRA^+/+^) or heterozygous (NPRA^+/−^), but not homozygous (NPRA^−/−^; 0 pups from 14 litters). Incisions along the left ventricular free wall were made to visualize EGFP signal on the inner septal surface and free wall of each ventricle from Cx40^egfp/+^/ NPRA^+/+^ and Cx40^egfp/+^/ NPRA^+/−^ pups (Fig. 6A,B). In the Cx40^egfp/+^/NPRA^+/+^ hearts, a broad network of EGFP^+^ fiber-like bundles were observed extending from the base of left ventricular septum that corresponded to the left bundle branch (LBB) (Fig. 6A). As the LBB extended toward the apex of the left ventricle, it gave rise to an extensive network of overlapping Purkinje fibers that formed a honeycomb like pattern within the left ventricular septal surface and free wall (Fig. 6A). Intriguingly, in several Cx40^egfp/+^/ NPRA^+/−^ pups examined, the individual fibers of the LBB appeared thinner and the overall Purkinje fiber network along the septal wall appeared far less dense compared to Cx40^egfp/+^/ NPRA^+/+^ littermates (Fig. 6B). Subsequently, the EGFP fluorescence was assessed in the ventricles from both groups by quantifying the green pixels using an image analysis method^28^. From these analyses, the Cx40^egfp/+^/NPRA^+/−^ ventricles revealed a significant reduction in the percentage of green pixels when compared to that in Cx40^egfp/+^/ NPRA^+/+^ ventricles (Fig. 6C; *P* < 0.005, 2-fold reduction).

### The effects of exogenous cGMP (8-Br-cGMP) on VCS gene expression and cardiomyocyte differentiation in embryonic ventricular cells

**Figure 7:**
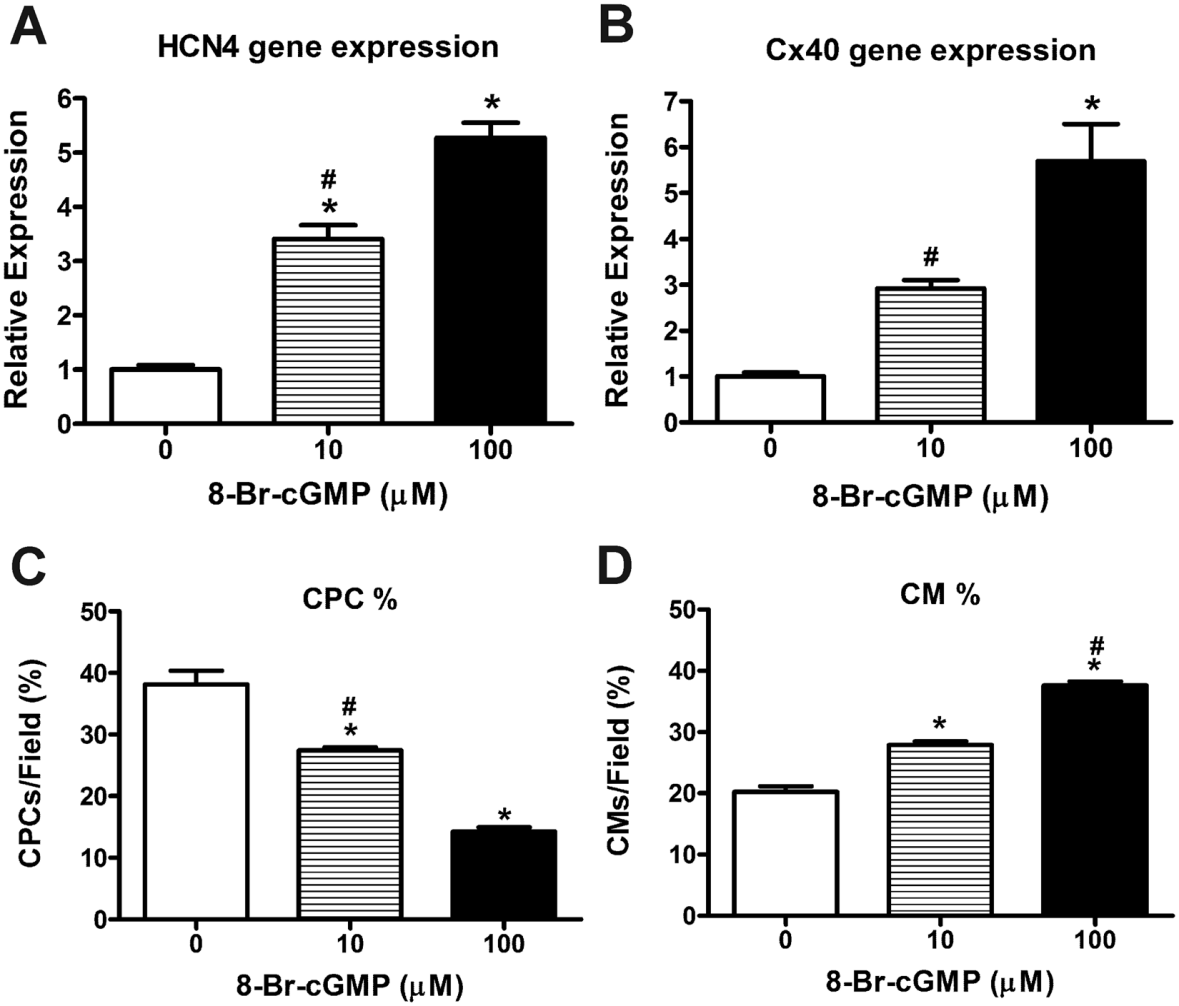
Effects of exogenous addition of 8-Br-cGMP on VCS marker gene expression and cell differentiation in E11.5 ventricular cell cultures: (**A**,**B**) RT-qPCR analysis was used to monitor HCN4 (A) and Cx40 (B) gene expression levels in cells treated with or without 8-Br-cGMP at indicated concentrations for 48 hours. Expression levels were normalized to GAPDH via ∆∆C_T_ method. Relative expression levels were presented as fold changes in comparison to the levels in untreated cultures (0 µM). N = 9 independent experiments per group, each bar represents mean ± SEM, *P < 0.005 Vs. Untreated or 0 µM, ^#^*P* < 0.005 Vs. 100 µM for panels A and B, One-way ANOVA with Tukey’s multiple comparisons post hoc test. (**C**,**D**) Exogenous addition of 8-Br-cGMP (10 and 100 µM) to E11.5 ventricular cell cultures derived from a Nkx2.5-Cre and ROSA-lacZ reporter system revealed significant differences in cellular differentiation of cardiac progenitor cells (CPC) and cardiomyocytes (CM) compared to untreated cultures (0 µM). N = 6 independent experiments. Each bar represents mean ± SEM. *P < 0.05 Vs. 0 µM for both CPC and CM percentages, ^#^*P* < 0.005 Vs. 10 µM for CM (D) or 100 µM for CPC (C), One-way ANOVA with Tukey post hoc test.

To determine if the classical NPRA/cGMP dependent pathway is critical for the regulation of HCN4 and Cx40 gene expression, an exogenous cell permeable cGMP compound (8-Br-cGMP) was added at 10 or 100 µM concentrations to E11.5 ventricular cells and VCS marker gene expression levels were determined (Fig. 7). Dosage of the 8-Br-cGMP was based on studies from other groups^29,30^ as well as reports which revealed that only a small fraction of exogenously added 8-Br-cGMP is detectable in the cytoplasm^31^. The addition of 8-Br-cGMP at 10 µM and 100 µM significantly increased the gene expression of HCN4 (3.4-fold and 5.3-fold respectively Vs. control, *P* < 0.005, Fig. 7A). Similarly, the addition of 8-Br-cGMP at 10 µM and 100 µM also increased the gene expression of Cx40 (2.9-fold and 5.7-fold respectively Vs. control, Fig. 7B). To examine the effects on ventricular cell differentiation, 8-Br-cGMP was added to E11.5 ventricular cells generated by crossing two knock-in mouse strains (Nkx2.5-Cre and ROSA-lacZ) as described in our previous studies. Using this approach, embryonic CPCs can be identified as cells positive for β-Gal but not MF20 (β-Gal^+^/MF20^-^) and CMs can be identified as cells positive for both markers (β-Gal^+^/MF20^+^)^14,16,32^. With the addition of 8-Br-cGMP to E11.5 cell culture, the percentage of CPCs significantly decreased with 10 µM and 100 µM doses compared to that of control cultures (1.4- and 2.7-folds respectively, *P* < 0.05, Fig. 7C). In contrast, addition of 8-Br-cGMP at both doses resulted in a significant increase in the percentage of CMs, Vs. control (1.4- and 1.9-folds respectively, *P* < 0.05, Fig. 7D).

### Effects of Rp-8-pCPT-cGMPS, a protein kinase G (PKG) inhibitor, on VCS gene expression in E11.5 ventricular cells treated with or without ANP

**Figure 8:**
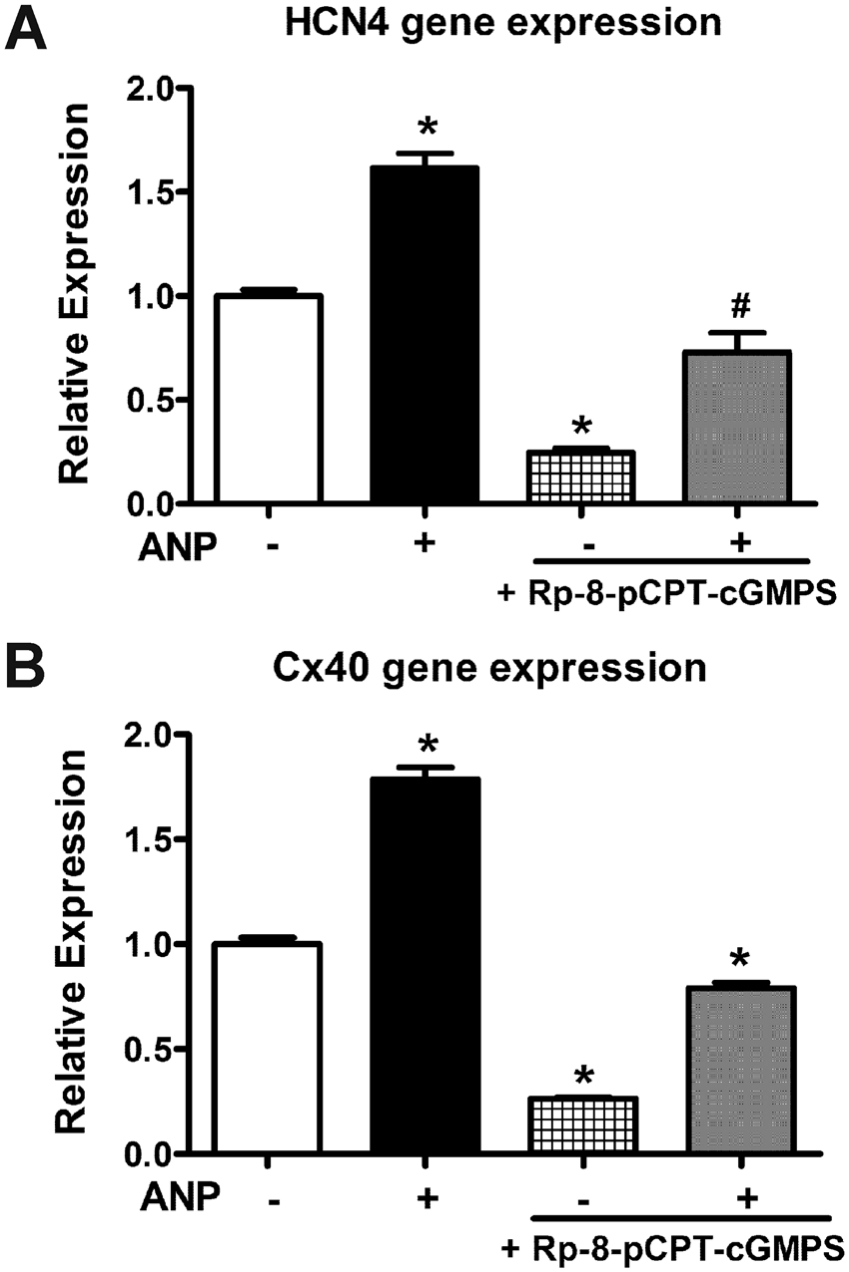
Gene expression of conduction system markers in E11.5 cultures treated with or without ANP and a PKG inhibitor, Rp-8-pCPT-cGMPS. RT-qPCR analysis was used to monitor HCN4 (**A**) and Cx40 (**B**) gene expression levels in cells treated with ANP (1 μg/ml) and or a PKG inhibitor, Rp-8-pCPT-cGMPS (100 µM) for 24 hours. Expression levels were normalized to GAPDH via ∆∆C_T_ method. Relative expression levels were presented as fold changes in comparison to the levels in untreated cultures (No ANP or PKG inhibitor). Addition of Rp-8-pCPT-cGMPS alone significantly reduced HCN4 and Cx40 gene expression. Co-treatment with ANP partially rescued the inhibitory effects of Rp-8-pCPT-cGMPS on VCS marker gene expression. N = 8 independent experiments per group, each bar represents mean ± SEM, *P < 0.005 Vs. all groups in both panels, ^#^*P* < 0.05 Vs. Cont (No ANP and No PKG inhibitor) and 0.005 Vs. all other groups in panel A. One-way ANOVA with Tukey’s multiple comparisons post hoc test.

Next, we tested the effects of a PKG inhibitor in E11.5 ventricular cells. A 100 µM dose of Rp-8-pCPT-cGMPS was added to E11.5 ventricular cell culture two times at a 12-hour interval for a period of 24 hours in the presence or absence of ANP (Fig. 8). HCN4 expression was significantly reduced upon addition of PKG inhibitor alone or in combination with ANP (4.2-fold and 1.4-fold respectively Vs. control, *P* < 0.05, Fig. 8A). Similarly, Cx40 expression was significantly reduced upon addition of this compound alone or in combination with ANP (3.8-fold and 1.3-fold respectively Vs. control, *P* < 0.05, Fig. 8B). Based on these results, it appears that ANP treatment can partially rescue the inhibitory effects of Rp-8-pCPT-cGMPS on VCS maker gene expression. Overall, these results suggest that ANP/NPRA/cGMP/PKG signaling axis is involved in the regulation of HCN4 and Cx40 gene expression in embryonic ventricular cells.

### Effects of exogenous ANP and NPRA genotype on microRNAs involved in the regulation of HCN4 and Cx40 gene expression

**Figure 9:**
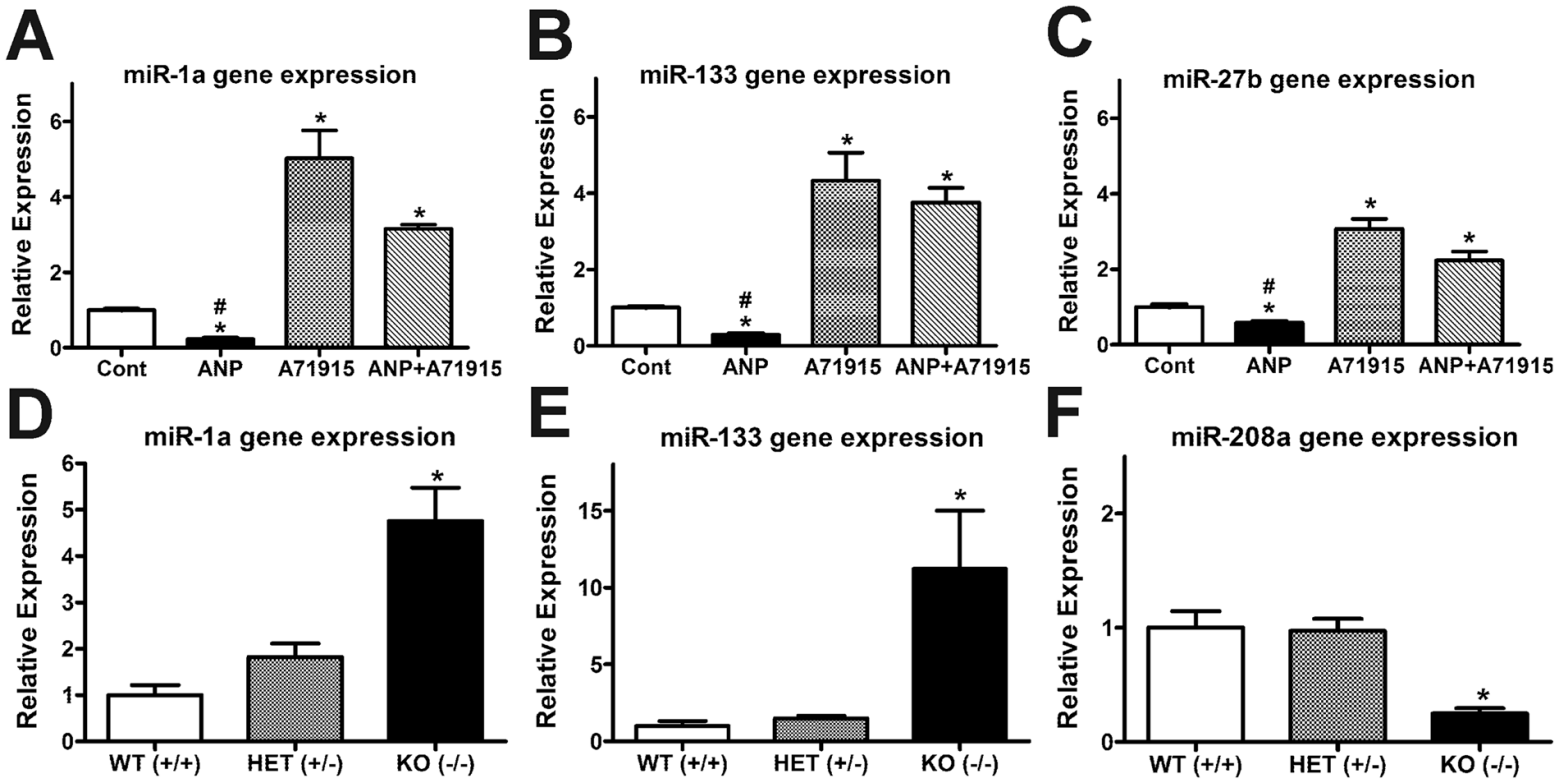
Effects of ANP/NPRA signaling and NPRA genotype on candidate microRNA levels in embryonic ventricles. (**A–C**) Expression analysis of miR-1a (A), miR-133 (B) and miR-27b (C) was performed on total microRNA isolated from E11.5 ventricular cell cultures treated with or without ANP (1 μg/ml) and NPRA inhibitor, A71915 (1 µM). MiR-1 and miR-133 target HCN4 mRNA and miR-27b regulates Cx40 mRNA levels. For all three miRNAs analyzed, addition of ANP significantly reduced expression of microRNAs, whereas addition of A71915 increased expression of microRNAs. N = 7 independent experiments. *P < 0.005 Vs. Cont, ^#^*P* < 0.005 Vs. ANP or ANP + A71915, One-way ANOVA with Tukey’s post hoc test. (**D–F**) Expression analysis of miR-1a (D), miR-133 (E) and miR-208a (F) was also performed on total microRNA isolated from E14.5 ventricles of NPRA wild type (WT), heterozygous (HET) and knockout (KO) mice. Compared to WT mice, NPRA KO mice demonstrated upregulation of miR-1a (D) and miR-133 (E) and downregulation of miR-208a (F). There were no significant differences in miR-27b expression between genotypes. For all panels, microRNA expression levels were normalized using U6 levels via ∆∆C_T_ method. Each bar represents mean ± SEM. N = 4 independent experiments. *P < 0.05 Vs. WT and HET, One-way ANOVA with Tukey’s post hoc test.

Recent studies have identified a regulatory role for microRNAs (miRNA or miR) in decreasing the levels of HCN4 (miR-1a and miR-133^33–35^) and Cx40 (miR-27b^36^) in cardiac tissue. However, it is not known whether ANP plays any role in the regulation of HCN4 and Cx40 gene expression via miRNA regulation in embryonic ventricular cells. Thus, expression analysis of the miR-1a, -133 and -27b was performed by RT-qPCR upon administration of ANP (1 μg/ml) and A71915 (1 µM) to E11.5 cell cultures for 48 hrs (Fig. 9A–C). The addition of ANP significantly reduced the expression of 1a, 133, and 27b Vs. control by 5-fold, 3.3-fold, and 1.6-fold, respectively (Fig. 9A–C). In contrast, the addition of A71915 significantly increased expression of all three miRNAs tested (5.0-fold, 4.3-fold, 3.1-fold, respectively, *P* < 0.005, Fig. 9A–C). The combination of ANP and A71915 treatment also significantly increased miRNA expression of 1a, 133 and 27b Vs. ANP alone and the control group (3.2-fold and 2.2-fold, *P* < 0.005) but not compared to A71915 alone (Fig. 9A–C).

We subsequently determined whether ablation of the NPRA receptor could regulate expression of miR-1a, -133, and -27b in E14.5 ventricles (Fig. 9D,E). Compared to WT ventricles, homozygous NPRA-KO ventricles demonstrated upregulation of miR-1a and −133 (4.8-fold and 11.2-fold, *P* < 0.05, Fig. 9D,E). For miR-27b expression, there were no statistical differences between any of the three genotypes (*P* = NS). Subsequently, expression of miRNA 208a was also analyzed, as it has been found to be essential for the sustained expression of Cx40 by another group^37^. At E14.5, homozygous NPRA-KO mice had significant downregulation of miR-208a compared to WT or heterozygotes (4-fold, *P* < 0.005, Fig. 9F). Collectively, these results suggest that gene expression of HCN4 and Cx40 in embryonic ventricular cells is regulated by ANP/NPRA signaling axis in part via significant changes in miRNA candidates that are known to control the stability of the respective target mRNAs^33–37^.

## Discussion

Despite the knowledge on the regulatory role of ANP in fluid homeostasis and systemic blood pressure during postnatal life^8^, it is not known whether ANP plays a direct role in cardiac development. Studies performed on NPRA KO mice indicated a decrease in neonatal survival at weaning due to heart abnormalities such as mesocardia and dextrocardia or fetal hydrops^38,39^. Surviving NPRA null mice develop cardiac hypertrophy, fibrosis and hypertension suggesting an antihypertrophic role for this peptide in heart development^25,40,41^. Recently, it has been suggested that ANP may play a local paracrine role in regulating the balance between CPC proliferation and differentiation through NPRA/cGMP signaling in mid-gestation ventricles^14^. Given the partial development of VCS components in E11/12 stage mouse hearts^42^ and the persistence of ANP expression throughout Purkinje fiber development^10,13,43^, we reasoned that ANP may be involved in the Purkinje cell formation and maturation in mid-gestation ventricles. HCN4 channels generate the pacemaker current (I_f_) in the heart. HCN4 is expressed in all components of the conduction system by E14.5^4^. Similarly, the gap junction protein Cx40 is also expressed in all components of conduction system by E14.5 excluding the SA node and the outer portion of the AV node. After birth, both HCN4 and Cx40 are expressed in the Purkinje fibers^18,44^, and thus serve as excellent markers of VCS development. Thus, we focused on the effects of ANP treatment and pharmacological or genetic ablation of NPRA signaling on VCS marker gene expression (HCN4 and Cx40) as well as VCS arborisation in this study.

We have previously reported that ANP treatment of E11.5 ventricular cells did not have any effect on the cell cycle activity of MF20 positive cardiomyocytes. However, ANP treatment significantly decreased the tritiated thymidine incorporation in MF20 negative and Nkx2.5+ CPCs^14^. Consistent with our earlier results, ANP treatment of E11.5 ventricular cells in this study significantly decreased the percentages of HCN4+ or Cx40+ cells that are negative for MF20 and increased the ratios of VCS marker positive CMs. These results suggest that ANP/NPRA signaling plays a critical role in the differentiation of HCN4+ or Cx40+ non-CM cells. Although some of these VCS marker positive non-CM population may represent other cell types such as endothelial cells or smooth muscle cells, we previously reported that E11.5 ventricular cell cultures treated with or without ANP did not contain any CD31 positive endothelial or α-smooth muscle actinin positive cells^14^. Since ANP treatment can significantly decrease cell cycle activity of E11.5 CPCs but not that of CMs^14^, it is likely that the increases in HCN+ or Cx40+ CMs in the present study with ANP treatment is most likely due to the differentiation of CPCs into VCS marker positive CMs. This notion is further supported by the previously demonstrated ability of E11.5 CPCs to differentiate into working CMs and conduction system cells^5^ as well as bipotential nature of trabecular cells in clonal analysis experiments^15^. Furthermore, a hypoplastic phenotype of Purkinje fibers in the NPRA knockout ventricles as well as gene expression changes in ventricular cells treated with or without ANP and A71915 are consistent with a pro-differentiation role for ANP in the VCS development.

Changes in VCS marker gene expression in response to ANP treatment could be attributed to multiple mechanisms including transcriptional, post-transcriptional and translational mechanisms. The promoter regions for HCN4 and Cx40 genes were cloned and sequenced^45,46^, however, further work needs to be done in this area to determine the role of ANP in transcriptional regulation of HCN4 and Cx40 promoter regions. Both pharmacological inhibition of NPRA, and genetic ablation of the NPRA receptor, resulted in upregulation of miR-1a and −133. While pharmacological inhibition of NPRA led to upregulation of miR-27b in primary cultures, there were no significant differences in miR-27b levels between NPRA KO and WT ventricles. MiR-1 and miR-133 are known to destabilize HCN4 mRNA^33–35^ while miR-27b is known to target Cx40 mRNA^36^. Moreover, miR-27b levels were shown to be significantly upregulated from E12.5 to E18.5 stages of ventricular development and miR-27b expression was localized to developing myocardium with no expression in the endocardium at E10.5^47^. Notably, Cx40-positive Purkinje fiber arborisation is known to increase with a concomitant decrease in the trabecular expression of Cx40 by the E16.5 stage^18,24^. It is possible that the regional differences in miR-27b (myocardium > endocardium) may facilitate robust expression of Cx40 in Purkinje fibers located in the endocardium while limiting the Cx40 expression in the trabecular myocardium.

Our results related to downregulation of miR-208a levels in NPRA KO hearts are consistent with a role for this miRNA in VCS development as suggested by another study, which found that miR-208a KO mice develop conduction deficits due to reduced Cx40 gene expression^37^. However, the molecular mechanisms underlying miR-208a mediated regulation of Cx40 expression and the potential link to VCS components are yet to be identified^37^. Cardiac miR-208a levels were shown to be significantly downregulated during atrial fibrillation^48^ and various forms of cardiomyopathy in end-stage heart failure patients^49,50^. In contrast, no significant changes in miR-208a levels were reported in mouse hearts subjected to hypertrophy via transverse aortic banding (TAB) after 21 days^50^. Notably, miR-208a KO mice subjected to TAB were resistant for cardiac hypertrophy and fibrosis^50^, whereas miR-208a cardiac specific transgenic mice developed significantly enlarged hearts even in the absence of any stress signals^37^. More recently, mechanical stretch of neonatal rat cardiomyocytes was shown to increase the levels of miR-208a and pretreatment of cultures with antagomir-208a led to significant decreases in stretch associated changes in hypertrophic gene program^51^. However, additional studies are required to confirm whether miR-208a levels are altered in other models of cardiac hypertrophy induced by angiotensin II or catecholamines. Since both ANP and BNP expression levels are significantly increased during cardiac hypertrophy as part of fetal gene expression program^52^, it would be interesting to examine the relationship between miR-208a expression levels and changes in VCS marker gene expression as well as CPC proliferation in future studies using relevant models of cardiac hypertrophy. Several studies linked miR-1a and −133 to cardiac development and regulation of HCN4 gene expression^33,35,53,54^. Notably, homozygous miR-1 KO mice exhibited 50% lethality by weaning age due to conduction deficits and ventricular septal defects^54^. Collectively, these reports support the notion that miR expression changes induced by ANP/NPRA signaling may in turn regulate VCS marker gene expression in embryonic ventricular cells.

Brain natriuretic peptide (BNP), a related family member of natriuretic peptides, is known to bind with membrane bound guanylyl cyclase receptors (NPRA and NPRB) as well as NPRC receptors with varying affinities^8^. BNP is also expressed in the mid-gestation stage ventricles albeit at lower levels compared to the levels of ANP^52,55^. In contrast, the expression pattern of BNP becomes more pronounced in E16.5 ventricles compared to that of ANP^55^. Notably, the immunoreactivity of ANP clearly exceeds that of BNP in the Purkinje fiber network of postnatal ventricles^43^. Collectively, these studies suggest that ANP may play a significant autocrine or paracrine role in the induction of VCS, while BNP may play a regulatory role in the expansion and maturation of VCS network at later stages of ventricular development. Indeed, BNP treatment was shown to increase proliferation and differentiation of CPCs via NPRB receptors in neonatal and adult hearts under both *in vitro* and *in vivo* conditions^56,57^. However, additional studies are required to confirm whether BNP plays any role in the differentiation of either CPCs or CMs into VCS cells in the embryonic and postnatal hearts. While our findings suggest a major role for NPRA receptors in the ANP induced VCS gene expression changes, ANP is also known to bind with NPRB and NPRC at a lower affinity. It is unlikely that ANP increases VCS gene expression through NPRB receptors since the maximal concentration used for ANP in this study (1 μg/ml; ~300 nM) is far below the half-maximal concentration (25 µM) that was required for ANP/NPRB mediated cGMP production in cell culture studies^58^. Although NPRC receptors are present in E11.5 ventricles, previous studies indicated that ANP treatment fails to decrease cAMP levels in these cells via NPRC pathway^14^. Taken together, these observations suggest that NPRB or NPRC signaling pathways may not be responsible for ANP mediated effects on VCS gene expression in our experiments.

Inhibition of NPRA receptors via shRNA approach was shown to induce cell cycle arrest, apoptosis and autophagy in gastric cancer cells^59^. We have previously reported that ANP treatment of E11.5 ventricular cells did not have any effect on the levels of apoptosis either in CPCs or cardiomyocytes^14^. In the present study, we did not find any significant changes in the total cell number in cultures treated with either ANP or A71915 (NPRA blocker). Additional studies are required to assess whether treatment of embryonic ventricular cells with ANP or absence of NPRA signaling can alter the levels of autophagy and if there is any direct relationship between autophagy and ANP/NPRA induced changes in VCS marker gene expression. Although endogenous cGMP levels were significantly increased in E11.5 ventricular cells stimulated with ANP via NPRA pathway, it is possible that effects of ANP on VCS marker gene expression may be attributed to cGMP-independent signaling pathways as reported in other studies^60^. Such alternative pathways were not pursued in this study since additional experiments using cell permeable 8-Br-cGMP or a PKG inhibitor further confirmed the role of ANP/NPRA/cGMP/PKG pathway in the regulation of HCN4 and Cx40 gene expression. However, we cannot rule out the involvement of cGMP independent pathways in ANP mediated regulation of miRNAs in embryonic ventricular cells. This notion is further supported by a report which showed that ANP but not S-nitroso-N-acetylpenicillamine (cGMP/PKG activator) treatment can lead to downregulation of miR-27b in human aortic smooth muscle cells^61^. Thus, additional studies are required to validate the role of cGMP dependent and independent pathways in ANP mediated miRNA regulation in the embryonic heart.

While additional electrophysiological and global gene expression studies are required to validate the physiological nature of ANP-induced VCS cells, we provided evidence that ANP treatment can increase the gene expression of the important VCS markers (HCN4 and Cx40) in embryonic ventricular cells through the NPRA/cGMP/PKG signal transduction pathway. Genetic ablation of NPRA can significantly decrease VCS marker gene expression and lead to defects in Purkinje fiber arborisation. These results provide new insights into the molecular mechanisms that guide development of the VCS and thus may facilitate the development of new therapeutic strategies for congenital heart defects associated with conduction abnormalities.

## Materials and Methods

### Animal maintenance, mouse strains and genotyping

All animal procedures were performed according to the Canadian Council on Animal Care guidelines and were approved by the Dalhousie University Committee on Laboratory Animal Care (Protocol No. 16-048). CD1 and C57BL/6 (BL6) mice were obtained from Charles River Laboratories (Montreal, Canada). Generation of the NPRA KO mouse strain was previously described^25^. In NPRA KO mice, exon 1 and intron 1 of the *Npr1* gene which encodes for the NPRA receptor were replaced with a neomycin resistance cassette. The Cx40^egfp^ mouse strain was utilized to study the formation of the Purkinje fiber network; in this strain, an enhanced green fluorescent protein (EGFP) coding sequence followed by pgk-neo cassette was inserted in frame at the Cx40 start codon^24^. Generation of the Nkx2.5-Cre mice was previously described^62^. These were engineered to have an internal ribosomal entry sequence (IRES) and a Cre-recombinase (Cre) coding sequence inserted into the 3′ untranslated region of the Nkx2.5 gene. The Rosa-lacZ reporter strain was obtained from Jackson Laboratories (Bar Harbor, Maine, USA). All knock-in and knock out lines were maintained in C57BL/6 (BL6) background. For genotyping, genomic DNA was extracted from ear punch biopsies and PCR amplification assay was performed using RedExtract amplification kit (Sigma, St. Louis, USA) and appropriate primer sets for each mouse line (see Suppl. Table S2). Female mice were mated with males and noontime on the day when the copulation plug was found was designated as embryonic (E) day 0.5 (E0.5). Unless otherwise stated, CD1 mice were used for all experimental procedures.

### Embryonic ventricular cell cultures

Timed pregnant females were anesthetized using 4% isoflurane and were sacrificed by cervical dislocation. Embryos were isolated from the uterine horns, followed by removal of the placenta using a Leica MZ16F stereomicroscope (Leica Microsystems, Richmond Hill, Ontario, Canada). Whole hearts were dissected out of embryos and the atria and outflow tracts were removed. Right and left ventricles from each embryo were placed in 0.2% v/v type I Collagenase (Worthington Biochemical Corp., Lakewood, New Jersey, USA) in PBS and incubated for 30 minutes at 37 °C to digest ventricular tissues. Following the 30-minute incubation period, tissue was triturated using a 200 µl pipette tip to mechanically dissociate cells from remaining tissue pieces. Cells were centrifuged at 4,000 rpm for 4 minutes, collagenase was discarded, and the pellet was neutralized with two washes of DMEM (Dulbecco’s Modified Eagles Medium; Wisent, Saint Bruno, Quebec, Canada) containing 10% fetal bovine serum (FBS; Wisent, Saint Bruno, Quebec, Canada). Unless otherwise noted, all cell cultures were treated with 10% FBS-DMEM. The number of cells was determined using a hemocytometer and cells were re-suspended in 10% FBS-DMEM to achieve the required cell numbers. Cells were plated at various densities on fibronectin (Sigma) coated 2- or 4-well chamber slides (250,000 cells/well, Nunc, Rochester, New York, USA), 35 mm dishes (500,000 cells/dish, Corning, New York, USA), or black-walled clear bottom 96-well plates (4000-100,000 cells/well) Greiner Bio-One, North Carolina, USA). Human embryonic kidney epithelial cells (HEK293) cells were purchased from American Type Culture Collection (ATCC, Virginia, USA) and maintained in 10% FBS-DMEM.

### Drug treatments and dosage protocols

ANP (Bachem, King of Prussia, Pennsylvania, USA Cat#: H-2100) or NPRA antagonist A71915 (Bachem, Cat#: H-3048) stock solutions were prepared by dissolving 0.5 mg of the compound in 0.5 ml of sterile H_2_O (Ambion, USA). The exogenous cGMP compound, 8-Bromoguanosine 3′,5′-cyclic monophosphate sodium salt (8-Br-cGMP**;** Sigma, Cat#: B1381) was prepared as a 100 mM stock solution and added to embryonic ventricular cell cultures at a final concentration of either 10 µM or 100 µM. The PKG inhibitor compound, Rp-8-pCPT-cGMPS (Tocris Bioscience, Cat#: 5524), was prepared as a 1 mM stock solution and a final concentration of 100 µM was added to the cells. Stock solutions for all drug compounds were aliquoted and stored at −20 °C and working solutions were prepared immediately prior to use on the day of the experiment.

Acutely isolated ventricular cells were maintained 10% FBS-DMEM for approximately 20 hours prior to starting any treatments. For experiments involving CD1 embryonic ventricular cell cultures treated with ANP and/or A71915, cells were treated every 12 hours over a 48-hour period. The exogenous cGMP compound 8-Br-cGMP and the PKG inhibitor compound Rp-8-pCPT-cGMPS, were added to embryonic cell cultures, two times at a 12-hour interval over a 24-hour period. Cx40^egfp/+^ whole embryos were cultured and immediately given a single dose of ANP (1 μg/ml) and/or A71915 (1 µM) for 24 hours. For measurement of intracellular cGMP, cells were treated with ANP and/or A71915 for a total period of 2 hours prior to performing the competitive immunoassays. Control cultures in all experiments received appropriate vehicle treatment (water or media) to match with the conditions used in treatment groups.

### Immune cytochemistry

Following drug treatment, embryonic ventricular cell cultures were fixed with 4% w/v paraformaldehyde (pH 7.4) for 5 min at room temperature, and were then permeabilized in 0.1% v/v Triton X-100 (Sigma) for 4 min. Following this, cultures were covered in blocking buffer solution [10% v/v goat serum (Gibo), and 1% w/v bovine serum albumin (BSA; Thermo Fisher Scientific) in PBS] for 1 hour at room temperature. After 1 hour, blocking buffer solution was removed and replaced with blocking buffer containing primary antibodies of choice, whose concentrations are listed in Suppl. Table S3, for 1 hour at room temperature. Slides were then washed with PBS three times for 3 min each, and were then incubated with secondary goat anti-mouse antibody conjugated to Alexa Fluor 488 (1:200, Invitrogen) and goat anti-rabbit antibody conjugated to Alexa Fluor 555 (1:200, Invitrogen) in blocking buffer for 1 hour. Nuclei were counterstained by immersion of a solution containing 1 µg/ml Hoechst 33258 (Sigma) in PBS. Slides were mounted with 0.1% propyl gallate (Sigma) solution [(0.1% w/v propyl gallate and 50% v/v glycerol (Thermo Fisher Scientific) in PBS] and examined using the Leica DM2500 fluorescence microscope and images were then captured using a Leica DFC 500 digital acquisition system.

### Total RNA extraction from cells and tissues

Cultured embryonic ventricular cells were lysed directly in 35 mm culture dishes by using TRIzol reagent (Invitrogen) method as described earlier^14,22,32^. For tissue, approximately 20 ventricles (left and right) from E11.5 embryos were collected and pooled from multiple time-pregnant females to obtain sufficient quantity of tissue and then were subjected to RNA extraction using the TRIzol method. Ventricles from embryos at later developmental stages or postnatal stages (neonatal) were minced into smaller pieces (collected from 1–3 ventricles, left and right) and RNA extraction was performed using TRIzol method as described earlier^14,22,32^. RNA content was quantified by measuring absorbance at 260 nm and 280 nm using a spectrometer (SmartSpec^TM^ Plus, Bio-Rad, Mississauga, Ontario, Canada). To ensure a high level of RNA purity, only samples with 260:280 ratio >1.8 were used in subsequent gene expression experiments. Samples meeting these standards of quality control were then immediately converted into more stable cDNA sequences using a SuperScript VILO kit (Invitrogen) and stored at −20 °C for real time quantitative PCR (RT-qPCR) gene expression analysis experiments. All RT-qPCR reactions were performed using an ECO thermocycler (Illumina, San Diego, California, USA) for 40 cycles: 15 sec at 95 °C, 60 sec at 60 °C. Once the amplification cycles were complete, melt curves were generated to confirm the amplification of a single primer product, with an extra cycle using the following conditions: 15 sec at 95 °C, 15 sec at 60 °C and 15 sec at 95 °C. RT-qPCR analysis was performed on a minimum of 3–6 independent RNA extractions (biological replicates) for each treatment group or as specified in the figure legends. RT-qPCR reactions were performed in duplicate wells for each biological replicate. All gene expression findings were normalized to GAPDH using the ΔΔC_T_ method^21^ and presented as fold changes relative to controls.

### MiRNA analysis

MiRNA was isolated from cultured cells or ventricular tissue using RNAzolRT reagent (GeneCopoeia, Rockville, MD, USA) and the samples were reverse transcribed using the All-in-One RT-qPCR kit (GeneCopoeia, Cat#: QP016) according to the supplier’s instructions. The cDNA samples were further processed through quantitative RT-qPCR using miRNA specific primer sets for 1a, 133, 27b, or 208a from GeneCopoeia. MiRNA expression for RT-qPCR analysis was normalized to a reference primer (U6) using the ΔΔC_T_ method. A thermal profile was utilized as follows: 50 °C for 2 min, 95 °C for 10 min, 95 °C for 10 sec, 63 °C for 20 sec, 72 °C for 10 sec; 40 cycles; then 95 °C, 60 °C and 95 °C for 15 sec each, using an ECO thermocycler (Illumina, San Diego, CA).

### Imaging of Cx40^egfp/+^ whole embryo cultures and neonatal ventricles

Timed pregnancies were set up between male Cx40^egfp/egfp^ and female C57/BL6 mice to generate Cx40^egfp+/−^ heterozygote whole embryos at E10.5, which were then cultured in 10% FBS-DMEM without adding any antibiotic or antimycotic agents. Immediately upon culture, ANP (1 μg/ml) and/or A71915 (1 µM) were added to culture media for 24 hours. After 24 hours, now at E11.5, whole embryos were imaged for detection of green fluorescence, and then hearts were removed and imaged for detection of green fluorescence using a Leica MZ16F stereomicroscope (Leica Microsystems, Richmond Hill, Ontario, Canada). Areas occupied by green pixels from digitized images were quantified using a previously described color subtractive image analysis method^28^. The percentage (%) of green pixel was determined for the combined ventricles of each heart using the following formula: Area occupied by green pixels (%) = [Green pixel area/(Green pixel area + area devoid of green pixels)] × 100.

Male and female mice double heterozygous for both NPRA KO allele and Cx40^egfp^ knock-in allele (NPRA-KO^+/−^/Cx40^egfp/+^) were crossed to generate neonates to visualize arborisation of the Purkinje fiber network. Genotypes of 1-day old neonatal pups (ND1s) were determined using tail biopsies as described earlier. Whole hearts were removed from neonates and imaged for visualization of green fluorescence. Areas occupied by green pixels were quantified as described earlier.

### Second messenger assays for cGMP

To measure cGMP concentrations in cultures treated with ANP and or A71915, competitive immunoassays were performed using the two-step protocol of the cGMP HTRF assay kit (Cisbio, Cat#: Cat#: 62GM2PEB) as described earlier^14,32^ with minor modifications. For these assays, 100,000 cells were seeded per well in 384 well plates (Greiner Bio-One) initially. The fluorophores were excited at a wavelength of 337 nm and emission was detected at 665 nm and 620 nm using a POLARstar Omega plate reader (BMG Labtech). Results were calculated using the 665 nm/620 nm ratio and expressed as Delta F values using data reduction steps described in manufacturer’s instructions. The cGMP standard curves were generated by plotting the Delta F values from standards with known cGMP concentrations. Subsequently, cGMP concentrations in experimental samples were determined by interpolating their corresponding delta F values from the standard curves.

### Statistical analysis

Data are presented as mean ± standard error of the mean (SEM). A two-tailed unpaired t-test was utilized to compare means between two groups. Multiple group comparisons were analyzed by ANOVA and Tukey multiple comparison post hoc tests. Significance for all analyses was assigned at *P* < 0.05. For each experiment, the number of experiments/replicates is displayed in the corresponding figure legends. All statistical analysis was performed using GraphPad Prism software.

### Data availability statement

All data generated or analysed during this study are included in this manuscript and supplementary data information.

## Electronic supplementary material

Supplementary Information
