# Supplementary material for: Characterizing the role of atrial natriuretic peptide signaling in the development of embryonic ventricular conduction system
Source: Sci Rep. 2018 May 2;8:6939. doi: 10.1038/s41598-018-25292-0 (PMC5932026; doi:10.1038/s41598-018-25292-0)
Supplement: Supplementary file 1 — Supplementary Information [file 41598_2018_25292_MOESM1_ESM.pdf]

Supplementary information

Characterizing the role of atrial natriuretic peptide signaling in the development of embryonic ventricular conduction system

Arun Govindapillai, Adam Hotchkiss, Mark Baguma-Nibasheka, Robert A. Rose, Lucile Miquerol, Oliver Smithies, Nobuyo Maeda and Kishore B.S. Pasumarthi

Supplementary Table S1: List of primers and corresponding primer sequences for real-time quantitative PCR (RT-qPCR) experiments and expected amplicon sizes:

Name of the Primer	Primer Sequence (5' to 3')	Expected Amplicon Sizes (bp)
Cx40-F<br>Cx40-R	CAGAGCCTGAAGAAGCCAAC<br>GACTGTGGAGTGCTTGTGGA	137
HCN4-F<br>HCN4-R	CCTCCTGCGCCTCTTGAGGCTTT<br>TGCCAATGAGGTTCACGATGCGT	119
GAPDH-F<br>GAPDH-R	TCGTCCCGTAGACAAAATGG<br>TTGAGGTCAATGAAGGGGTC	132

F: Forward or sense primer; R: Reverse or antisense primer

Supplementary Table S2: List of primers utilized for genotyping and their expected amplicon sizes (bp):

Name of the primer	Primer Sequence (5’ to 3’)	Wild type allele	Knock-in/knockout allele
**Nkx2.5-S**<br>**Nkx2.5-AS**<br>**Cre-S**	GCCCTGTCCCTCGGATTTCACACC<br>ACGCACTCACTTTAATGGGAAGAG<br>GATGACTCTGGTCAGAGATACCTG	264	583
**Rosa 1**<br>**Rosa 2**<br>**Rosa 3**	AAAGTCGCTCTGAGTTGTTAT<br>GCGAAGAGTTTGTCCTCAACC<br>GGAGCGGGAGAAATGGATATG	650	320
**Npr1-S**<br>**Npr1-AS**<br>**Neo-AS**	GCATGGTTCAGCTCTAAGA<br>CTAACCCTGTGAACTGTAAGC<br>CCTTCAGTTATCTACATCTGC	339	500
**Cx40-S**<br>**Cx40-AS**<br>**Neo-AS**	CTCCAATTAACTCCTTGTGAGCC<br>AGGCTGAATGGTATCGCACC<br>CTTGCCGAATATCATGGTGG	380	450

Supplementary Table S3: List of primary antibodies and corresponding dilutions used for immunostaining experiments.

Primary Antibody	Dilution	Source/Catalogue #
ß-galactosidase (Rabbit polyclonal)	1:50	Chappel ICN Catalogue #: 55976
Connexin40 (Cx40) (Rabbit polyclonal)	1:50	Alpha Diagnostics Catalogue #: Cx40-A
HCN4 (Rabbit polyclonal)	1:50	Alamone Laboratories Catalogue #: APC-052
Sarcomeric myosin (MF20) (Mouse monoclonal)	1:50	Developmental Studies Hybridoma Bank, Catalogue #: MF-20
